# Lidocaine could promote the cuproptosis through up-regulating the long noncoding RNA DNMBP-AS1 in Hep-2 cells

**DOI:** 10.1186/s12885-025-13533-1

**Published:** 2025-01-22

**Authors:** Wei Liu, Yi Yu, Yi He, Meihong Lv

**Affiliations:** 1 Department of Otolaryngology, the Second Affiliated Hospital of Dalian Medical University, Dalian, Liaoning China; 2 General Surgery, the First Affiliated Hospital of Dalian Medical University, Dalian, Liaoning China; 3 Department of Urology, the Second Affiliated Hospital of Dalian Medical University, 467, Zhongshan Road, Shahekou District, Dalian, 116044 Liaoning China; 4 Department of Anesthesiology, the First Affiliated Hospital of Dalian Medical University, 222, Zhongshan Road, Xigang District, Dalian, 116011 Liaoning China

**Keywords:** Lidocaine, Hep-2 cells, Cuproptosis, IncRNA DNMBP-AS1

## Abstract

**Background:**

Lidocaine is a traditional local anesthetic, which has been reported to trigger apoptosis through the mitochondrial pathway, independent of death receptor signaling. Cuproptosis is a copper triggered mitochondrial cell death mode. In this study, we explored the biological effects of lidocaine on cuproptosis in Hep-2 cells and studied the relevant mechanisms.

**Methods:**

quantitative RT-PCR was used to measure the expression level of long noncoding RNA (IncRNA) DNMBP-AS1. DNMBP-AS1 siRNA (si-DNMBP-AS1) were transfected into Hep-2 cells to verify the roles of DNMBP-AS1 in cuproptosis. 24 h treatment with 20 nM elesclomol and 2 µM CuCl_2_ was performed to promote the occurrence of Cuproptosis. Cell proliferation, migration and apoptosis assays ware utilized to analyze biological effect of lidocaine and DNMBP-AS1 on Hep-2 cells. Active caspase-3 were also determined after treatment.

**Results:**

DNMBP-AS1 was significantly upregulated during cuproptosis in Hep-2 cells. The si-DNMBP-AS1 significantly increased the cell viability with nonactivated caspase-3, promoted the cell migration and suppress the cuproptosis. Lidocaine was cytotoxic to the Hep-2 cells in a dose- and time-dependent manner. Exposure to 10 µM of lidocaine for 24 h did not reduce the viability or activated the caspase-3, but significantly increased the expression of DNMBP-AS1, and promote the cuproptosis. Anymore, si-DNMBP-AS1 reversed the pro-cuproptosis function of lidocaine.

**Conclusions:**

lidocaine was cytotoxic to Hep-2 cells in a time- and dose-dependent manner, promoted the cuproptosis through up-regulating DNMBP-AS1. The results of this study offered initial optimism that lidocaine could be used in an adjuvant or neoadjuvant fashion in cancer treatment.

## Introduction

Cuproptosis is a new cell death mode, which is related to copper and mitochondrial respiration [1–3]. The pathological mechanism is that copper directly interacts with the fatty acylated components of the tricarboxylic acid (TCA) cycle, resulting in excessive aggregation of fatty acylated proteins and loss of iron sulfur cluster proteins, thereby stimulating proteotoxic stress and cell death. Recent studies have found that copper cell death is closely related to human cancer [4–6], proving that cuproptosis is closely related to the occurrence and development of cancer.

Long non-coding RNA (LncRNA) play a key role in gene regulation because they can affect cell proliferation, migration and genome stability. In recent years, transcriptome sequencing has revealed that thousands of aberrantly expressed LncRNA are associated with different cancers [7, 8]. The mechanisms of action of LncRNA in carcinogenesis include enhancing chromatin state and methylation, maintaining the stability of proteins or protein complexes, and acting as sponges for inhibiting microRNA (miRNA). Previous studies have shown that lncRNA DNMBP-AS1 is significantly associated with poor overall survival in patients with breast cancer and colon cancer [9, 10]. Yin et al. found that DNMBP-AS1 could promote prostate cancer development by regulating LCLAT1A [11]. Although these studies confirmed its oncogenic function, recent study reported that DNMBP-AS1 could inhibit the progression of hepatocellular carcinoma through cuproptosis induction [12].

Lidocaine is a kind of amide local anesthetic. It has been found that lidocaine has good potential in anti-tumor treatment [13, 14]. A previous study demonstrated that lidocaine induces apoptosis through the mitochondrial pathway, independent of death receptor signaling [15].

In this study, we explored the biological effect of lidocaine on Hep-2 cells and the related mechanism of cuproptosis, which is expected to provide more theoretical references for the treatment of cancer.

## Methods

### Cell culture and treatment

The Hep-2 cell lines were purchased from ATCC (American Type Culture Collection). The cells were cultured in Dulbecco’s modified Eagle’s medium (DMEM; Gibco Corporation, USA) with 10% fetal bovine serum (HyClone, USA) and 1% penicillin/streptomycin (Invitrogen) at 37 °C with 5% CO_2_. To evaluate the effect of lidocaine, we seeded the Hep-2 cells at a density of 4000 cells/well in 96-well plates in triplicate. Once they reached 80% confluence, the cells were treated for 24 h, 48 h and 72 h with various concentrations of lidocaine (1 µM, 5 µM, 10 µM, 25 µM, 50 µM and 100 µM) and compared with a control. For cuproptosis occurrence, cells were treated with 20 nM elesclomol and 2 µM CuCl_2_ for 4 h, 12 h and 24 h [16].

### Cell transfection

si-DNMBP-AS1 and negative control (si-ctrl) were synthesized by GenePharma (Shanghai, China). Hep-2 cells were uniformly plated in 96 well plates. When the cells reached about 80–90% fusion, they were transfected with plasmid by Lipo3000 (Invitrogen, Carlsbad, CA, USA). Cells were harvested for the subsequent experiments following incubation at 37 °C for 48 h.

### Quantitative real-time polymerase chain reaction (qRT − PCR)

Trizol (Beyotime, Shanghai, China) was used to extract the total RNA from LUAD cells and tissues. RNA was reverse-transcribed into complementary DNA (cDNA) using SuperScript VILO cDNA Kit (Thermo Fisher Scientific, Inc.). The primer sequences for PCR were as follows: DNMBP-AS1 forward, 5′-TCGCTCCTCATAGCGAGTCT-3′ and reverse, 5′-TGACTTCCTTATCCGCTCCC-3′; and GAPDH forward, 5′-GGAGCGAGATCCCTCCAAAAT-3′ and reverse, 5′-GGCTGTTGTCATACTTCTCATGG-3′. The real-time PCR was performed with a SYBR Green PCR kit (Takara Biotechnology Co., Ltd., Dalian, China) to measure the expression of DNMBP-AS1 and GAPDH. The data were analyzed according to the 2^−△△CT^ method [17].

### Cell proliferation assay

96 well plates were used to seed Hep-2 cells (2 × 10^5^ cells / well). After incubation at 37 ° C and 5% CO_2_ for different times, the CCK-8 kit provided by Tiangen (Hangzhou, China) was mixed at 10 µ L / well, and cells were incubated for 3 h at 37 ° C and 5% CO_2_. Finally, we read the absorbance at 450 nm on the microplate reader (Thermo Fisher Scientific, Inc.) [17].

### Cell death analysis

Cells were seeded in six-well plates at the density of 1 × 10^5^ cells/well. Each well contained 2 ml of culture medium.Cells were incubated for 24 h and then were exposed to the anti-androgen media containing C-S serum and Bicalutamide for 24 h. Cells then were collected and rinsed with PBS and re-suspended in 300 µl of binding buffer. Next, 5 µl of PI was added to the cell suspensions and incubated for 15 min in the dark. Cell death was then detected by using a BD FACSVerse flow cytometer [17].

### Enzyme-linked immunosorbent assay (ELISA)

The cleaved caspase-3 have bioactivity to induce apoptosis. The concentrations of active caspase-3 in Hep-2 cells after treatment were determined using human caspase-3 ELISA kit (Elabscience Biotechnology Co., Ltd., Hubei, China), according to the manufacturer’s instructions [18].

### Migration assay

Wound healing assay was used to detect the ability of migration of cell lines. The cells with concentration of 1 × 10^4^/ml were cultured in 6-well plate at 37 °C with 5% CO2 for 24 h and grown until 80% confluent. was added to culture for 24 h. A straight line scratch was made on the cells using a sterile 20 µL disposable serological pipette. The cells were washed with 1 ml median to remove debris and smooth the edge of the scratch and cultured in median without FBS for 12 h. Images of the cell proliferation were taken using a microscope [17].

### Statistical analysis

One-way analysis of variance (ANOVA) were used to assess differences between groups. SPSS 25.0 software was performed for statistical analyses. All experiments were performed independently and repeated three times. *P* < 0.05 was considered statistically significant.

## Results

### DNMBP-AS1 was significantly upregulated by exogenous introduction of copper ions

**Fig. 1:**
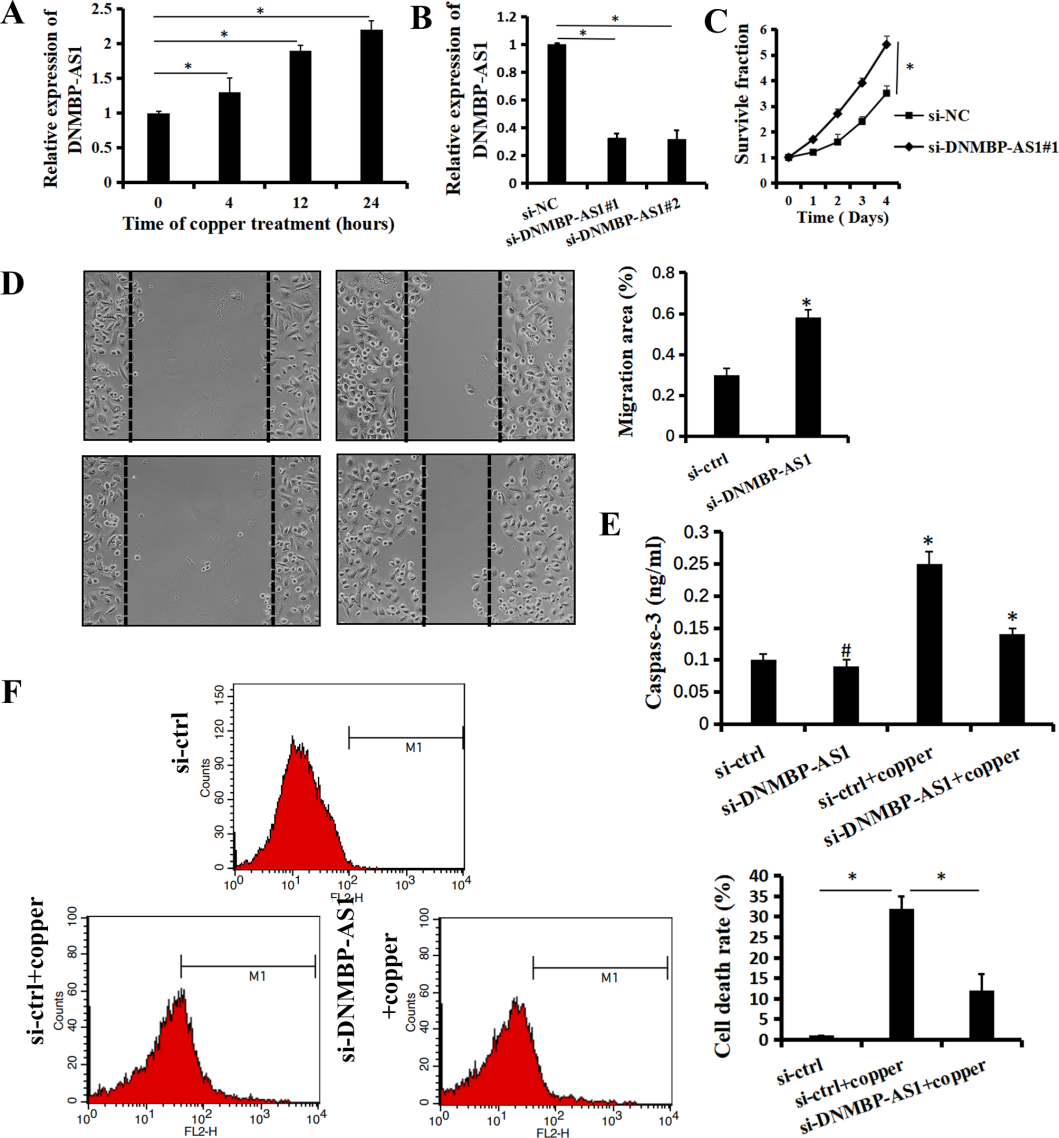
DNMBP-AS1 was significantly upregulated by exogenous introduction of copper ions. (**A**) Changes of the lncRNA DNMBP-AS1 expression in the constructed cuproptosis cell model was assessed by qRT-PCR. (**B**) Efficiency of DNMBP-AS1 knockdown (si-DNMBP-AS1-1 and si-DNMBP-AS1-2) was assessed by qRT-PCR. (**C**) Effect of si-DNMBP-AS1 on the proliferation of Hep-2 cells was detected by CCK-8 assays. (**D**) Migration of Hep-2 cells was detected via wound healing assay. (**E**) Concentration of active caspase-3 was analyzed by ELISA. (**F**) Cell death of Hep-2/si-ctrl and Hep-2/si-DNMBP-AS1 cells induced by exogenous introduction of copper ions were analyzed via flow cytometry assay. Data are shown as the mean ± SD of three independent experiments. *P < 0.01, ^#^*P* > 0.05

We first evaluated whether copper could affect the expression of the DNMBP-AS1. Elesclomol is a copper ionophore that shuttles copper into the cell. We treated Hep-2 cells with copper chloride (2µM) combined with elesclomol (20nM) for 24 h. The results of qRT-PCR showed that DNMBP-AS1 was significantly upregulated during cuproptosis (*p* < 0.05. Figure 1A). Then, CCK-8 assay showed that si-DNMBP-AS1 significantly increased the cell viability of Hep-2 cells (*p* < 0.05. Figure 1B, C). Anymore, si-DNMBP-AS1 greatly increased the migration ability of Hep-2 cells (*p* < 0.05. Figure 1D). Further analysis showed that si-DNMBP-AS1 significantly deactivated the caspase-3 in Hep-2 cells, and suppress the cuproptosis induced by copper treatment (*p* < 0.05. Figure 1E, F). These results demonstrated that DNMBP-AS1 could play a important role in cuproptosis in laryngocarcinoma.

### Lidocaine could promote the cuproptosis through up-regulating the DNMBP-AS1

**Fig. 2:**
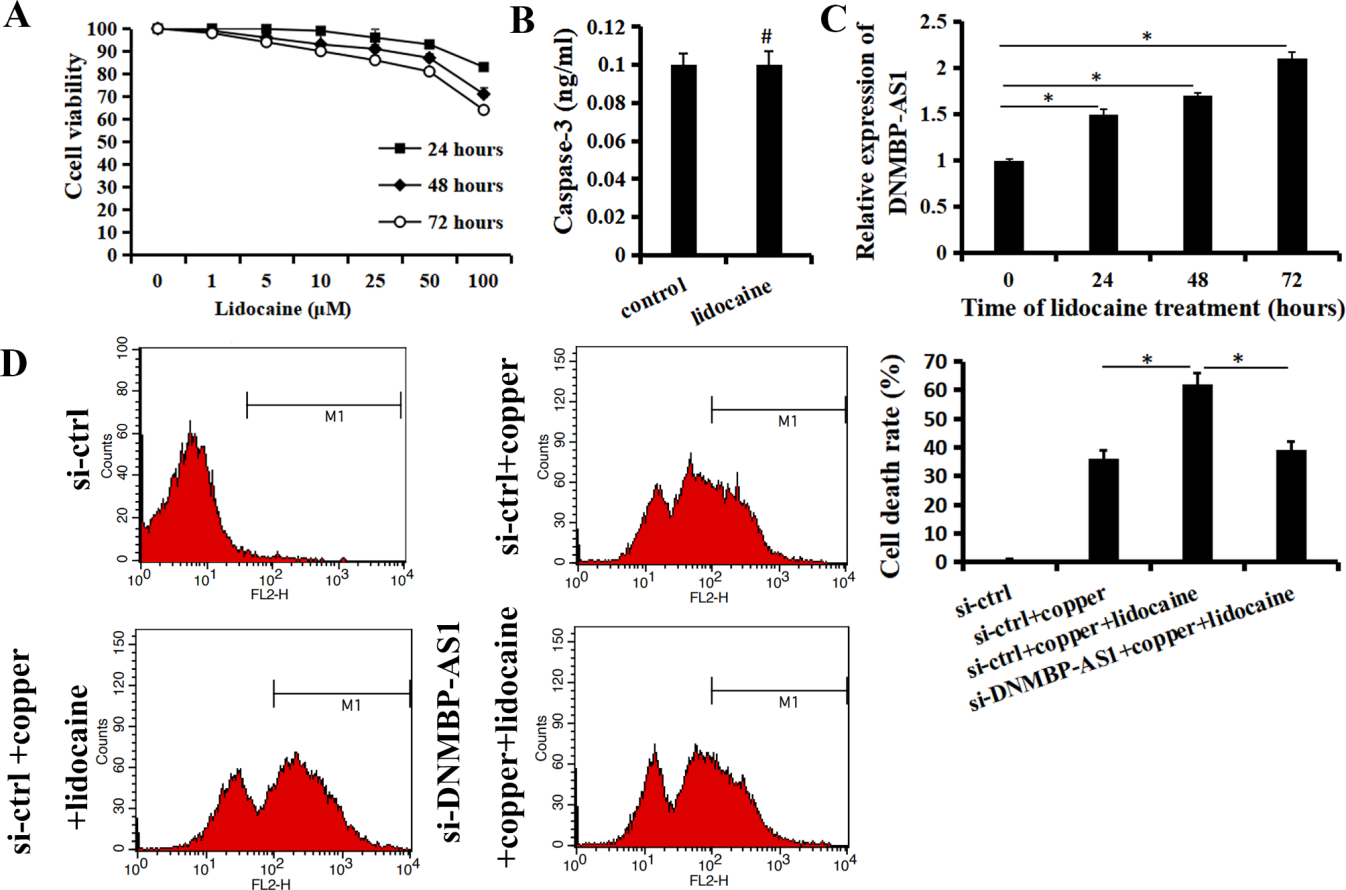
Lidocaine could promote the cuproptosis through up-regulating the DNMBP-AS1 (**A**) Viability of Hep-2 cells treated with lidocaine was assessed by CCK-8. (**B**) Concentration of active caspase-3 was analyzed by ELISA. (**C**) Effect of lidocaine on the DNMBP-AS1 expression was assessed by qRT-PCR. (**D**) effect of lidocaine on the cuproptosis induced by exogenous introduction of copper ions were analyzed via flow cytometry assay. Data are shown as the mean ± SD of three independent experiments. *P < 0.01, ^#^*P* > 0.05

CCK-8 assay showed that lidocaine was cytotoxic to the Hep-2 cells in a dose- and time-dependent manner (Fig. 2A). Interestingly, exposure to 10 µM of lidocaine for 24 h did not reduce the viability of Hep-2 cells or increased the concentration of active caspase-3 (*p* > 0.05. Figure 2B), but significantly increased the expression of DNMBP-AS1 (*p* < 0.05. Figure 2C). Cell apoptosis analysis showed that exposure to 10 µM of lidocaine for 24 h significantly promote the cuproptosis in Hep-2 cells (*p* < 0.05). Anymore, si-DNMBP-AS1 reverse the pro-cuproptosis function of lidocaine (*p* < 0.05. Figure 2D). The results indicated that lidocaine could promote the cuproptosis through up-regulating the DNMBP-AS1.

## Discussion

The concept cuproptosis was was proposed in March 2022. So far, there are limited reports on this issue in the literature [16]. The in-depth study of cuproptosis is helpful to promote the new understanding of tumor pathogenesis. The function and regulatory role of LncRNA in cuproptosis are increasingly recognized. Xie et al. constructed a risk model based on cuproptosis-related LncRNA can accurately predict the prognosis of cutaneous melanoma [19]. Liu et al. identified eight cuprotosis-related LncRNA signature of HNSC as promising biomarkers for predicting the benefit of head and neck squamous cell carcinoma from immunotherapy [20]. Xu et al. reported that cuproptosis-related lncRNA MIR31HG could act as an oncogene in lung adenocarcinoma [21]. A recent study reported that DNMBP-AS1 could partake in the cuproptosis induction [12]. In this study, we found that exposure to copper chloride combined with elesclomol significantly upregulated the expression of DNMBP-AS1 in Hep-2 cells. The si-DNMBP-AS1 significantly increased the cell viability, deactivated the caspase-3 and suppressed the cuproptosis induced by copper treatment, which indicated that DNMBP-AS1 could play a important role in cuproptosis.

Previous studies have shown that local anesthetics are toxic to multiple cancer types. Tu et al. reported that lidocaine could prevent breast cancer growth by targeting neuronatin to inhibit nerve fibers formation [22]. Xu et al. found that lidocaine could enhance the effects of chemotherapeutic drugs against bladder cancer [13]. Wan et al. showed that lidocaine could impede the tumor progression in hepatocellular carcinoma via circ-DYNC1H1/miR-520a-3p/USP14 axis [23]. In this study, we found that lidocaine had cytotoxic effect on Hep-2 cells in a dose- and time-dependent manner. Though exposure to 10 µM of lidocaine for 24 h did not reduce the viability of Hep-2 cells or activate the caspase-3, it significantly increased the expression of DNMBP-AS1, and enhanced the cuproptosis in Hep-2 cells, which was reversed by si-DNMBP-AS1. These results indicated that lidocaine could promote the cuproptosis through up-regulating the DNMBP-AS1. Stevens et al. found that death receptors were not involved in lidocaine-induced cell death, which could be mediated by the intrinsic mitochondrial death pathway [15]. Interestingly, cuproptosis has also been demonstrated as a copper dependent regulated form of cell death driven by aggregation of mitochondrial lipoacylated proteins [16].

## Conclusions

This study found that lidocaine was cytotoxic to human Hep-2 cells in a time- and dose-dependent manner, promoted the cuproptosis through up-regulating DNMBP-AS1. The results of this study are initially optimistic that lidocaine can be used as an adjuvant or neoadjuvant modality in the treatment of cancer, and further in vivo validation are required to evaluate therapeutic potential of lidocaine and its action mechanism in a more clinically relevant context.

## Data Availability

The datasets used and analysed during the current study are available from the corresponding author on reasonable request.

## References

[1] Tang D, Kroemer G, Kang R. Targeting cuproplasia and cuproptosis in cancer. Nat Rev Clin Oncol. 2024;21:370–88. 38486054 10.1038/s41571-024-00876-0

[2] Xie J, Yang Y, Gao Y, et al. Cuproptosis: mechanisms and links with cancers. Mol Cancer. 2023;22:46. 36882769 10.1186/s12943-023-01732-y PMC9990368

[3] Wang Y, Zhang L, Zhou F. Cuproptosis: a new form of programmed cell death. Cell Mol Immunol. 2022;19:867–8. 35459854 10.1038/s41423-022-00866-1 PMC9338229

[4] Hu Q, Wang R, Ma H, Zhang Z, Xue Q. Cuproptosis predicts the risk and clinical outcomes of lung adenocarcinoma. Front Oncol. 2022;12:922332. 36003780 10.3389/fonc.2022.922332 PMC9393616

[5] Song Q, Zhou R, Shu F, Fu W. Cuproptosis scoring system to predict the clinical outcome and immune response in bladder cancer. Front Immunol. 2022;13:958368. 35990642 10.3389/fimmu.2022.958368 PMC9386055

[6] Li L, Li L, Sun Q. High expression of cuproptosis-related SLC31A1 gene in relation to unfavorable outcome and deregulated immune cell infiltration in breast cancer: an analysis based on public databases. BMC Bioinformatics. 2022;23:350. 35996075 10.1186/s12859-022-04894-6 PMC9394027

[7] Zhan L, Li J, Wei B. Long non-coding RNAs in ovarian cancer. J Exp Clin Cancer Res. 2018;37:120. 29921308 10.1186/s13046-018-0793-4 PMC6008930

[8] Rao AKDM, Rajkumar T, Mani S. Perspectives of long non-coding RNAs in cancer. Mol Biol Rep. 2017;44:203–18. 28391434 10.1007/s11033-017-4103-6

[9] Gao S, Lu X, Ma J, Zhou Q, Tang R, Fu Z, Wang F, Lv M, Lu C. Comprehensive Analysis of lncRNA and miRNA Regulatory Network reveals potential prognostic non-coding RNA involved in breast Cancer progression. Front Genet. 2021;12:621809. 34220926 10.3389/fgene.2021.621809 PMC8253500

[10] Yang L, Yang T, Wang H, Dou T, Fang X, Shi L, Li X, Feng M. DNMBP-AS1 regulates NHLRC3 expression by sponging miR-93-5p/17-5p to inhibit Colon cancer progression. Front Oncol. 2022;12:765163. 35574307 10.3389/fonc.2022.765163 PMC9092830

[11] Yin X, Wang S, Ge R, Chen J, Gao Y, Xu S, Yang T. Long non-coding RNA DNMBP-AS1 promotes prostate cancer development by regulating LCLAT1. Syst Biology Reproductive Med. 2023;69(2):142–52. 10.1080/19396368.2022.2129520 36602957

[12] Zhang G, Sun J, Zhang X. A novel cuproptosis-related LncRNA signature to predict prognosis in hepatocellular carcinoma. Sci Rep. 2022;12:11325. 35790864 10.1038/s41598-022-15251-1 PMC9256635

[13] Zhong Wang Q, Liu J, Lu J, Cao X-Y, Wang Y, Chen. Lidocaine promotes autophagy of SH-SY5Y cells through inhibiting PI3K/AKT/mTOR pathway by upregulating miR-145. Toxicol Res. 2020;9:467–73. 10.1093/toxres/tfaa049 PMC7467247 32905277

[14] Zhang C, Xie C, Lu Y. Local anesthetic lidocaine and Cancer: insight into Tumor Progression and Recurrence. Front Oncol. 2021;11:669746. 34249706 10.3389/fonc.2021.669746 PMC8264592

[15] Werdehausen R, Braun S, Essmann F, Schulze-Osthoff K, Walczak H, Lipfert P, Markus F. Stevens; Lidocaine Induces Apoptosis via the Mitochondrial Pathway Independently of Death Receptor Signaling. Anesthesiology; 107:136–143 (2007). 10.1097/01.anes.0000268389.39436.66 17585225

[16] Xu M, Mu J, Wang J, Zhou Q, Wang J. Construction and validation of a cuproptosis-related lncRNA signature as a novel and robust prognostic model for colon adenocarcinoma. Front Oncol. 2022;12:961213. 35965536 10.3389/fonc.2022.961213 PMC9367690

[17] Xu X, Wang W, He Y, et al. Prognostic marker VPS72 could promote the malignant progression of prostate cancer. BMC Cancer. 2024;24:713. 38858662 10.1186/s12885-024-12488-z PMC11163694

[18] Cong L, Liu X, Bai Y, et al. Melatonin alleviates pyroptosis by regulating the SIRT3/FOXO3α/ROS axis and interacting with apoptosis in atherosclerosis progression. Biol Res. 2023;56:62. 38041171 10.1186/s40659-023-00479-6 PMC10693060

[19] Zhou Y, Shu Q, Fu Z, Wang C, Gu J, Li J, Chen Y, Xie M. A novel risk model based on cuproptosis-related lncRNAs predicted prognosis and indicated immune microenvironment landscape of patients with cutaneous melanoma. Front Genet. 2022;13:959456. 35938036 10.3389/fgene.2022.959456 PMC9354044

[20] Yang L, Yu J, Tao L, Huang H, Gao Y, Yao J, Liu Z. Cuproptosis-related lncRNAs are biomarkers of prognosis and Immune Microenvironment in Head and Neck squamous cell carcinoma. Front Genet. 2022;13:947551. 35938003 10.3389/fgene.2022.947551 PMC9354258

[21] Mo X, Hu D, Yang P, Li Y, Bashir S, Nai A, Ma F, Jia G, Xu M. A novel cuproptosis-related prognostic lncRNA signature and lncRNA MIR31HG/miR-193a-3p/TNFRSF21 regulatory axis in lung adenocarcinoma. Front Oncol. 2022;12:927706. 35936736 10.3389/fonc.2022.927706 PMC9353736

[22] Li B, Xu H, He C, Zou W, Tu Y. Lidocaine prevents breast cancer growth by targeting neuronatin to inhibit nerve fibers formation. J Toxicol Sci. 2021;46:329–39. 34193770 10.2131/jts.46.329

[23] Liu H, Cheng, Jing, Xu H, Wan Z. Lidocaine has antitumor effect on hepatocellular carcinoma via the circ_DYNC1H1/miR-520a-3p/USP14 axis. Open Life Sci. 2021;16:766–80. 34435133 10.1515/biol-2021-0072 PMC8354378

